# Supplementary material for: Rapid Naloxone Administration Workshop for Health Care Providers at an Academic Medical Center
Source: MedEdPORTAL. 2020 Feb 14;16:10892. doi: 10.15766/mep_2374-8265.10892 (PMC7062540; doi:10.15766/mep_2374-8265.10892)
Supplement: Supplementary file 1 — A. Naloxone Training Workshop PowerPoint.pptx B. Naloxone Trainer's Guide.docx C. Naloxone Training Video.mp4 D. Training Kit.docx E. Pre- and Postintervention Survey.docx [file mep-16-10892-s001.zip › _Educational_Summary_Report_10892.pdf]

MedEdPORTAL® The AAMC Journal of Teaching and Learning Resources

Original Publication Open Access

Rapid Naloxone Administration Workshop for Health Care Providers at an Academic Medical Center

Raagini Jawa, MD, MPH*, Thuy Luu, PharmD, BCPS, MPH, Melissa Bachman, PharmD, BCPS, Lindsay Demers, MS, PhD

*Corresponding Author: raagini.jawa@bmc.org

Abstract

Introduction: Opioid overdose is a growing problem in the US. Often, residents are first responders to community and in-hospital opioid overdoses, and so, hands-on naloxone administration education is necessary. While residents get a brief algorithm on suspected opioid overdose during their mandatory American Heart Association basic life support training, there is a lack of hands-on standardized curricula on how to administer this lifesaving medication. **Methods:** To fill this gap, we developed a hands-on workshop for medical trainees on how to respond to an opioid overdose. Trainees who completed our workshop left with a first-responder naloxone kit using the Massachusetts statewide open prescription. All attendees were asked to take a voluntary pre- and posttraining survey. **Results:** A total of 80 trainees from a variety of specialties and training levels participated in this workshop. We were able to successfully link the pre- and postdata of 29 participants. Trainees were assessed on comfort in administering naloxone as a first responder and in teaching patients how to administer naloxone (via a 5-point Likert scale) and on percentage of time they prescribed naloxone to high-risk patient populations. We saw statistically significant increases in comfort in using naloxone and comfort in teaching patients to administer naloxone. **Discussion:** This innovative curriculum provides an adaptable, short, and effective workshop with hands-on practice for medical trainees at a variety of training levels. The workshop can efficiently train future health care professionals how to approach an opioid overdose.

Keywords
Opioid Overdose, Nasal Naloxone, Naloxone, Harm Reduction, Interdisciplinary Medicine, Interprofessional Education, Substance Abuse/Addiction, Internal Medicine, Opioids

Educational Objectives

By the end of this workshop, learners will be able to:

1. Identify the signs and symptoms of opioid overdose.
2. Describe the mechanism of how nasal naloxone reverses an opioid overdose.
3. Administer single-step and two-step nasal naloxone via hands-on demonstration.
4. Advocate for appropriate prescribing of naloxone to high-risk patients and first responders.

Introduction

In 2017, the U.S. Department of Health and Human Services declared the opioid crisis a public health emergency.[1] Opioid overdose deaths were six times higher in 2017 compared to 1999, with over 47,000 deaths attributed to opioid overdose in 2017.[1] Although the overall opioid national prescribing rate has been declining since 2012 (81.3 prescriptions per 100 persons in 2012 versus 66.5 prescriptions per 100 persons in 2016), the amount of illicitly made fentanyl continues to rise, further contributing to opioid overdose deaths.[1] In response to the opioid crisis, the Department of Health and Human Services has focused on five major priorities: better addiction prevention and treatment, better targeting of overdose reversing drugs, better data, better pain management, and better research.[1]

Citation:
Jawa R, Luu T, Bachman M, Demers L. Rapid naloxone administration workshop for health care providers at an academic medical center. *MedEdPORTAL*. 2020;16:10892.
https://doi.org/10.15766/mep_2374-8265.10892

Naloxone nasal spray is indicated for the emergency treatment of known or suspected opioid overdose with respiratory or central nervous system depression.[2] In 2015, the Food and Drug Administration approved a prefilled, single-dose intranasal spray containing 4 mg of naloxone hydrochloride in 0.1 ml.[2] Between 2010 and 2014, the number of states expanding naloxone access to individuals including at-risk patients, relatives, and first responders increased from 16 to 30.[3] There have been several observational studies looking at the impact of opioid

Copyright © 2020 Jawa et al. This is an open-access publication distributed under the terms of the Creative Commons Attribution license.

overdose education in reduction of opioid overdose death rates. In one such study conducted in 19 Massachusetts communities, overdose education and naloxone rescue kits were given to 2,912 potential bystanders, who reported 327 rescues with naloxone.[4]

Previously, overdose education and naloxone distribution targeted high-risk patients who used opioids or their family members. In 2018, Dr. Jerome Adams, the U.S. Surgeon General, released an advisory encouraging education on naloxone use for bystanders such as health care practitioners, family and friends of people with opioid use disorder, and community members at the front line for these high-risk patients.[5] However, evidence suggests that medical providers not only are underprescribing[6] but lack comfort with opioid overdose prevention techniques.[7] A survey of emergency medicine providers showed a lack of time, a lack of training, and a lack of knowledge in willingness to prescribe naloxone.[8] While trainees (residents and fellows) are given a brief algorithm on suspected opioid overdose during their American Heart Association basic life support (BLS) training, there remains a lack of standardized hands-on curricula on how and when to administer naloxone.

According to an opioid summary by the National Institute on Drug Abuse, Massachusetts is in the top 10 states with the highest rates of opioid overdose deaths, twofold higher than the national rate.[9] In 2017, there were 2,054 opioid-related deaths in the state; 30% were from the county and neighboring county of our institution.[10] Given the significant number of opioid overdose deaths in our community and the lack of a standard curriculum for providing opioid overdose education for trainees in our residency, we developed a program to meet this need. The goal of this program was to measure the impact of a 15-minute overdose education training workshop led by volunteer internal medicine residents, pharmacists, and public safety officers on self-efficacy and comfort of residents at an urban health center. A unique aspect of our workshop was that, using the Massachusetts statewide open prescription, it expedited providing a naloxone kit to trainees who completed the session.

This workshop fills a unique gap in the available medical education curricula as it is associated with bystander naloxone distribution and can be accomplished in 15 minutes. A search of *MedEdPORTAL* using the keyword *naloxone* identified three unique publications: One includes naloxone training within a larger multiday substance use disorder training session for students,[11] another discusses an adverse event for pulmonary edema after naloxone administration,[12] and the third addresses indications for naloxone for inpatient opioid reversal.[13] While there is literature on the importance of naloxone training for medical trainees,[14] our curriculum is unique in that it is a stand-alone brief workshop whose primary focus is on naloxone education for health care providers. Even though the initial curriculum was developed for residents and fellows, it is generalizable to the medical community at large.

Methods

Our Institution

The workshop took place at Boston Medical Center (BMC), a 567-bed urban tertiary care academic institution in Boston, Massachusetts, affiliated with Boston University School of Medicine (BUSM). All evaluation procedures were approved and deemed exempt by the BUSM Institutional Review Board. The graduate medical education department supports over 800 residents and fellows ranging across all medical and surgical specialties. Our institution serves the surrounding community, which includes a culturally, socially, and economically diverse population. At the time of the workshop, all BMC trainees were mandated to complete BLS training.

Program Details

Our initial pilot cohort was the internal medicine intern class of 2017 to whom we provided a 5-minute presentation during intern orientation. This presentation included how to recognize and respond to an opioid overdose, how naloxone works to reverse the effects of opioids, and the differences between two-step naloxone and one-step naloxone. This was followed by a 10-minute hands-on demonstration by our pharmacist trainers on how to assemble and administer single-step and two-step naloxone as a first responder. During this time, the interns practiced assembling both formulations, asked questions about the curriculum, and filled out an open prescription to obtain naloxone as a first responder. As this was a pilot implementation, we gathered informal, formative feedback on workshop effectiveness and how to improve the training.

Program Expansion and Details

Following our pilot training, we expanded the workshop to include all graduate medical trainees and faculty at BMC with four additional 3-hour training workshops in the fall of 2017. The target audience was all BMC medical/surgical residents and fellows and BUSM/BMC faculty. Recruitment for the workshops was via department-wide email invitations, electronic advertisements, and flyer distribution throughout the medical campus. At the drop-in workshops, we had four to six workshop trainers per hour who trained groups of 10 residents/fellows at a time. Trainers consisted of volunteer BMC internal medicine

Copyright © 2020 Jawa et al. This is an open-access publication distributed under the terms of the Creative Commons Attribution license.

residents, emergency medicine residents, public safety officers, Project ASSERT (Alcohol & Substance Abuse Services, Education and Referral to Treatment) staff, and inpatient pharmacists. Participants had the opportunity to obtain a naloxone kit as a first responder, utilizing the open prescription for naloxone under the prescriptive authority of Dr. Alexander Walley, the medical director of the Opioid Overdose Prevention Pilot Program at the Massachusetts Department of Public Health (MA DPH).

Simultaneously, the Massachusetts Medical Society and the MA DPH devised an educational platform around issues of safer prescribing practices and substance use disorder by identifying 10 core competencies for residents and fellows to complete.[15] We developed a partnership with the BMC graduate medical education department in which attending the naloxone workshop allowed residents and fellows to fulfil one of the competency requirements.

Implementation of Naloxone Training Curriculum

The target audience for this curriculum included residents, fellows, faculty, and nonclinicians. We took an experiential learning approach so that learners could combine knowledge along with psychomotor skills in knowing when and how to administer naloxone. It was assumed that participants had no preexisting knowledge of naloxone use for opioid overdose; however, all participants had completed BLS certification prior to attending the workshop.

Equipment/Environment

This training was best run in any classroom or hall where there were several tables for trainers and trainees to be seated in small groups. The equipment necessary for the training included a printed version of Appendix A, which served as a handout on recognizing an overdose and reviewed how to administer naloxone. Additional materials would include a single-step and a two-step naloxone demonstration kit, if available (Appendix A, slide 5, shows both demonstration kits; Appendix D shows another view of the single-step kit).

Personnel

Trainers were volunteer pharmacists, public safety officers, and internal medicine residents. We used a multidisciplinary approach to training; however, all trainers were equally qualified to lead the workshop. Prior to the workshop, each trainer attended a brief review of the curriculum described in Appendices A and B led by a clinical pharmacy specialist. This training required one volunteer trainer per table of five to 10 trainees. Using Appendix B as a guide, the trainer led a discussion of the 5-minute curriculum and conducted a 5-minute hands-on demonstration of how to administer the two types of naloxone as a first responder. Appendix C serves as a sample training video for future trainers' reference or for programs that may not have live trainers.

Evaluation

We used a pre/post survey design (Appendix E) to evaluate the efficacy of this program. We had three primary outcome measures: (1) comfort administering naloxone as a first responder, (2) comfort teaching patients how to administer naloxone, and (3) percentage of time participants prescribed naloxone to patients with opioid addiction or high-risk IV drug use behaviors. Both comfort items were measured on a 5-point scale (1 = *extremely uncomfortable,* 5 = *extremely comfortable*). The percentage of time prescribing naloxone to patients was measured on a 4-point scale (0%-25%, 26%-50%, 51%-75%, and 76%-100%).

Anonymous electronic surveys soliciting participation in the program were sent approximately 1 month prior to implementation and were blinded using a unique identifier created by participants. At that time, we collected baseline data from potential participants on the three outcomes mentioned above. A total of 80 trainees from a variety of specialties and training levels participated in this workshop. Approximately 1 month after implementation, we sent out the same survey to all program participants, asking them to respond to questions on the same three outcomes. We then linked these 1-month postdata to the predata in order to make comparisons. In total, we were able to successfully link the pre- and postdata of 29 participants, 80% of whom were residents and 20% of whom were fellows.

Preintervention Survey Data

The preintervention survey showed 66% of trainees (residents and fellows) were prescribing less than 25% naloxone to patients with opioid use disorder or patients with high-risk behaviors including IV drug use. Despite attending required BLS instruction that mandated naloxone training and being at an institution very focused on harm reduction for patients with substance use disorder, only 37% of trainees felt moderately to extremely comfortable administering naloxone as a first responder, and just 18% felt moderately to extremely comfortable teaching their patients how to administer naloxone.

Results

To compare participant responses before and after participation, we used a series of nonparametric sign tests appropriate for Likert-type data. As shown in the Table, we saw statistically significant increases in comfort using naloxone as well as in comfort teaching patients to administer naloxone. We did not

Copyright © 2020 Jawa et al. This is an open-access publication distributed under the terms of the Creative Commons Attribution license.

Table. Descriptive Statistics and Results of Sign Tests ($n = 29$)

Item	Pretraining *M* (*SD*)	Posttraining *M* (*SD*)	Pre/Post Differences			*p*
			Negative	Tie	Positive	
Comfort administering naloxone as a first responder	2.6 (0.9)	4.2 (0.7)	1	3	25	<.001
Comfort teaching patients how to administer naloxone	2.3 (0.9)	4.1 (0.7)	1	2	26	<.001
Percent time naloxone is prescribed	1.9 (1.2)[a]	2.1 (1.2)[b]	5	16	8	>.500

[a]0%-25%.
[b]26%-50%.

see a statistically significant change among participants in the percentage of time naloxone was prescribed. However, we can think of three compelling reasons for this finding: It could be that 1 month was not adequate for participants to see enough patients with these issues to appreciably change the frequency at which they prescribed naloxone, especially given that we measured in 25% increments; that trainees who made the effort to attend were already prescribing naloxone at a high rate but lacked comfort with regard to the medication itself and thus sought out this additional training; or that trainees, despite increased comfort, did not actually change their prescribing behavior.

Discussion

We designed a multidisciplinary workshop focused on naloxone training for health care trainees. Although we presumed that our participants already had some baseline knowledge about naloxone, either from BLS or from the robust focus on substance use disorder at BMC, our results indicated that the workshop positively reinforced attendees to have increased self-efficacy in administering and teaching patients how to administer naloxone.

In our experience, aligning the workshop to meet one of the Massachusetts Medical Society's core competencies for prevention and management of prescription drug misuse allowed an increased number of trainees to undergo formal in-person naloxone training and served as an incentive.

A 5-minute didactic followed by a 10-minute hands-on demonstration allowed trainees to engage in an honest discussion of their preconceived notions about naloxone use and opioid overdoses, perceived barriers to naloxone use, and prescribing practices. By utilizing demonstration drug devices, the hands-on portion let each trainee learn how to assemble and administer both single-step and multistep naloxone as a first responder.

Given the simplicity of the curriculum, trainings can be conducted by anyone, including volunteer physicians, pharmacists, and nonclinical staff, which would incur no additional financial cost for small or large medical institutions. We used trainers from multiple disciplines who were able to initiate discussions on safe naloxone storage and use, hospital and community access, and examples of overdoses first responders could encounter. Pharmacist trainers provided the benefit of being able to answer medication-specific questions trainees had about naloxone. Public safety trainers provided real-world perspectives on safely responding to opioid overdoses. Physician trainers were able to emphasize the importance of prescribing to high-risk patients and reducing the stigma of substance use disorder.

We have adapted this curriculum to train large groups of hospital staff members from a variety of clinical and nonclinical backgrounds. We hosted the training workshop as multiple several-hour sessions to help accommodate schedules of working physicians and trainees; however, other institutions may choose to provide shorter workshops that can be completed over multiple days. With that in mind, we believe that this resource is adaptable and transferable to other health care education institutions and is a valuable tool for any first responder.

Via the open prescription, we were able to prescribe naloxone to interested first-responder trainees. In doing this, we aimed to reduce the stigma of carrying naloxone as a first responder and reinforced the Surgeon General's statement encouraging bystander naloxone education and use. Overall, our naloxone training workshop fostered a community focus on harm reduction and advocacy for hospital-wide naloxone trainings.

Limitations

This curriculum is limited as it is an isolated workshop. Several residents and fellows were unable to attend because of their individual hospital schedules despite our incentivizing participation to meet a core competency. While many participants attended the training and completed a preworkshop in-person survey, there was overall a lower response rate on the postsurvey as it was administered via email 1 month after the workshop.

Our results are limited in their scope as we surveyed trainees who attended, a disproportionate number of whom were from

Copyright © 2020 Jawa et al. This is an open-access publication distributed under the terms of the Creative Commons Attribution license.

the internal medicine department. While we can presume that trainees in other specialties had increased self-efficacy in naloxone administration, we cannot definitively make this conclusion. Given the nature of our survey evaluation, participation bias may have inflated our results. The lack of change in naloxone prescribing patterns may have been due to inadequate time for behavior change thanks to short postsurvey collection time or may have been affected by selection bias. Because we assessed impact only 1 month after training, the longitudinal impact of this educational intervention is unknown. Surveying these trainees 1 year after the intervention may provide more insight on how this training impacted their knowledge, prescribing patterns, and naloxone administration as first responders. Future iterations of the curriculum could include objective outcomes of change in naloxone prescribing for at-risk patients with opioid use disorder and coprescriptions with new opioids, as well as assessing change in frequency of opioid overdose education during patient encounters.

Limitations related to implementation of this curriculum may include health insurance coverage and access to an open prescription for naloxone. Additionally, the curriculum relies upon a large volunteer trainer workforce, which may not be available at all institutions. Implementing the curriculum requires adequate demonstration devices and supplies.

Conclusions and Reflections

Given the rising rates of opioid overdose deaths, there is an increasing public health need to train first responders how to approach an opioid overdose efficiently. Our innovative curriculum provides an adaptable, short, effective, and hands-on workshop for medical trainees. The curriculum can be delivered to small or larger groups of participants at a variety of training levels.

Reflecting on the implementation and evaluation of our workshop, we plan to continue to expand this program to all hospital nonclinical staff as well as the surrounding local community. Moving forward, we will adapt the hands-on naloxone training to include only the single-step naloxone, which is the preferred modality. In the future, we hope to incorporate a more robust survey to assess whether our intervention has an impact on medical trainees' prescribing practices or use of naloxone as first responders.

Our hope is that widespread use of this curriculum with medical trainee first responders will not only augment knowledge of how to respond to an opioid overdose but also increase comfort with naloxone use.

Appendices

A. Naloxone Training Workshop PowerPoint.pptx

B. Naloxone Trainer's Guide.docx

C. Naloxone Training Video.mp4

D. Training Kit.docx

E. Pre- and Postintervention Survey.docx

All appendices are peer reviewed as integral parts of the Original Publication.

Raagini Jawa, MD, MPH: Infectious Disease/Addiction Medicine Fellow, Department of Medicine, Boston Medical Center

Thuy Luu, PharmD, BCPS, MPH: Clinical Pharmacy Specialist—Internal Medicine, Boston Medical Center

Melissa Bachman, PharmD, BCPS: Clinical Pharmacy Specialist Lead—Internal Medicine, Boston Medical Center

Lindsay Demers, MS, PhD: Assistant Professor, Department of Medicine, Boston University School of Medicine; Director, Education Evaluation Core, Boston University School of Medicine

Acknowledgments

Many thanks to Dr. Jeff Schneider, Dr. Alex Walley, and Michael Botticelli for their guidance, mentorship, and review of our workshop and curriculum.

Disclosures

Thuy Luu is now an employee of Biogen.

Funding/Support

None to report.

Ethical Approval

The Boston University School of Medicine Institutional Review Board approved this study.

References

1. U.S. Department of Health and Human Services National Opioids Crisis website. https://www.hhs.gov/opioids. Updated March 6, 2018. Accessed April 14, 2018.
2. Narcan [package insert]. Radnor, PA: ADAPT Pharma, Inc; 2017.
3. Wheeler E, Jones TS, Gilbert MK, Davidson PJ. Opioid overdose prevention programs providing naloxone to laypersons—United States, 2014. *MMWR Morb Mortal Wkly Rep*. 2015;64(23): 631-635.
4. Walley AY, Xuan Z, Hackman HH, et al. Opioid overdose rates and implementation of overdose education and nasal naloxone

Copyright © 2020 Jawa et al. This is an open-access publication distributed under the terms of the Creative Commons Attribution license.

distribution in Massachusetts: interrupted time series analysis. *BMJ*. 2013;346(7894):f174. https://doi.org/10.1136/bmj.f174

5. U.S. Surgeon General's advisory on naloxone and opioid overdose. U.S. Department of Health and Human Services Office of the Surgeon General website. https://www.hhs.gov/surgeongeneral/priorities/opioids-and-addiction/naloxone-advisory/index.html. Updated April 5, 2018. Accessed February 1, 2019.
6. Guy GP Jr, Haegerich TM, Evans ME, Losby JL, Young R, Jones CM. Vital signs: pharmacy-based naloxone dispensing—United States, 2012–2018. *MMWR Morb Mortal Wkly Rep*. 2019; 68(31);679-686. https://doi.org/10.15585/mmwr.mm6831e1
7. Winograd RP, Davis CS, Niculete M, Oliva E, Martielli RP. Medical providers' knowledge and concerns about opioid overdose education and take-home naloxone rescue kits within Veterans Affairs health care medical treatment settings. *Subst Abus*. 2017;38(2):135-140. https://doi.org/10.1080/08897077.2017.1303424
8. Dwyer KH, Samuels L, Moore RL, et al. Physician attitudes and perceived barriers to prescribing nasal naloxone rescue kits in the emergency department. *Ann Emerg Med*. 2013;62(4)(suppl): S43. https://doi.org/10.1016/j.annemergmed.2013.07.400
9. National Institute on Drug Abuse. Massachusetts Opioid Summary. National Institute on Drug Abuse website. https://www.drugabuse.gov/node/pdf/21994/massachusetts-opioid-summary. Updated March 2019. Accessed October 28, 2019.
10. Massachusetts Department of Public Health. Number of opioid-related overdose deaths, all intents by county, MA residents: 2010-2018. Commonwealth of Massachusetts website. https://www.mass.gov/files/documents/2019/08/21/Opioid-related-Overdose-Deaths-by-County-August-2019.pdf. Published August 2019. Accessed October 28, 2019.
11. Monteiro K, Dumenco L, Collins S, et al. Substance use disorder training workshop for future interprofessional health care providers. *MedEdPORTAL*. 2017;13:10576. https://doi.org/10.15766/mep_2374-8265.10576
12. Keenan MP, Schenker KA, Sarsfield MJ. A complicated opioid overdose: a simulation for emergency medicine residents. *MedEdPORTAL*. 2017;13:10616. https://doi.org/10.15766/mep_2374-8265.10616
13. Lester P, Remolana R, Sandhu S, Scott J. Road map for opioid management in the inpatient setting: a structured approach to opioid selection and titration. *MedEdPORTAL*. 2016;12:10470. https://doi.org/10.15766/mep_2374-8265.10470
14. Taylor JL, Rapoport AB, Rowley CF, Mukamal KJ, Stead W. An opioid overdose curriculum for medical residents: impact on naloxone prescribing, knowledge, and attitudes. *Subst Abus*. 2018;39(3):371-376. https://doi.org/10.1080/08897077.2018.1439800
15. Core competencies. Massachusetts Medical Society website. http://www.massmed.org/Physicians/Residents-and-Fellows/Core-Competencies. Published May 4, 2017. Accessed February 6, 2019.

Received: April 29, 2019
Accepted: November 29, 2019
Published: February 14, 2020

Copyright © 2020 Jawa et al. This is an open-access publication distributed under the terms of the Creative Commons Attribution license.
