# Supplementary material for: Rapid Naloxone Administration Workshop for Health Care Providers at an Academic Medical Center
Source: MedEdPORTAL. 2020 Feb 14;16:10892. doi: 10.15766/mep_2374-8265.10892 (PMC7062540; doi:10.15766/mep_2374-8265.10892)
Supplement: Supplementary file 1 — A. Naloxone Training Workshop PowerPoint.pptx B. Naloxone Trainer's Guide.docx C. Naloxone Training Video.mp4 D. Training Kit.docx E. Pre- and Postintervention Survey.docx [file mep-16-10892-s001.zip › A. Naloxone Training Workshop PowerPoint.pptx]

## Slide 1
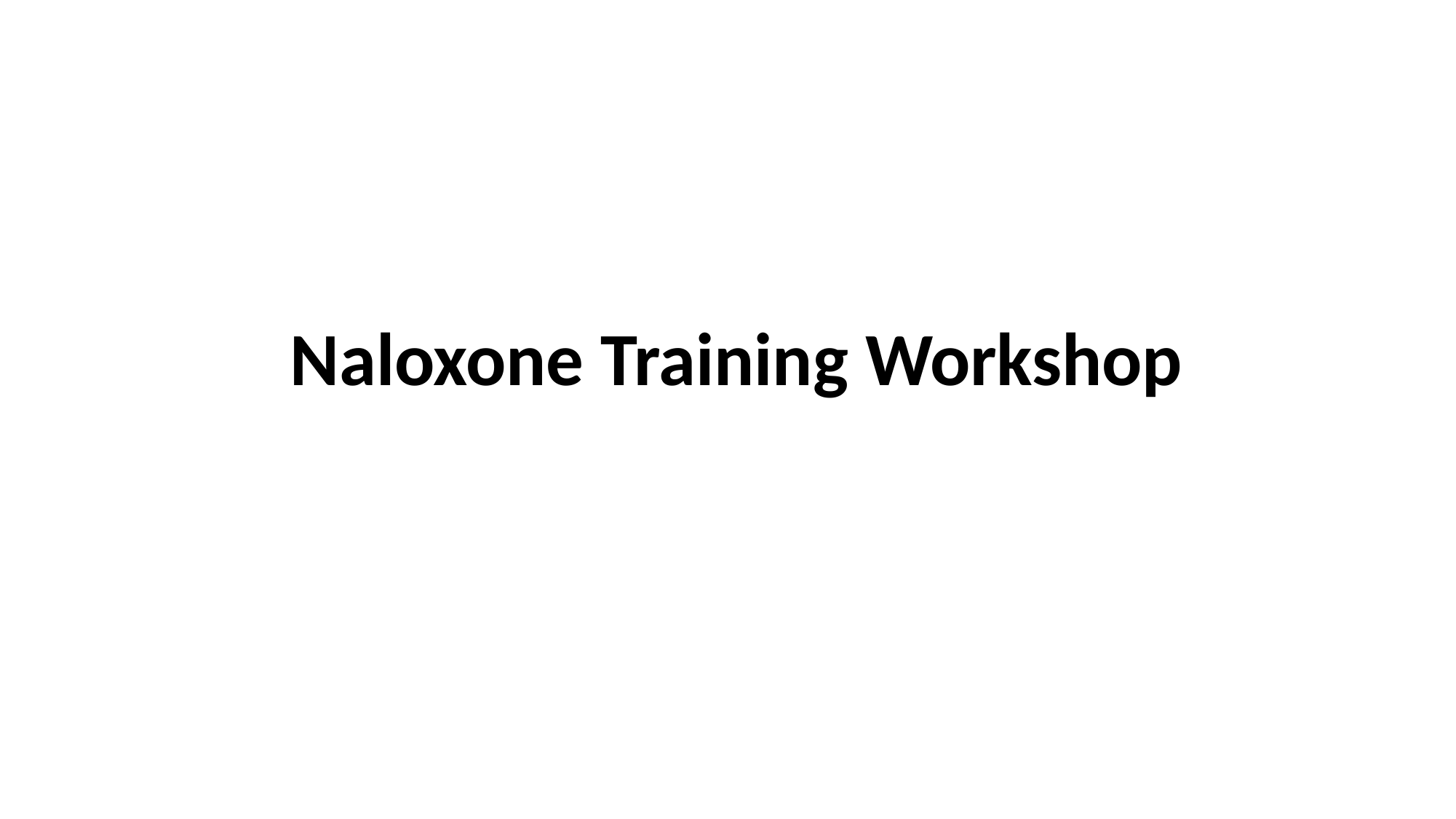

# Naloxone Training Workshop

## Slide 2
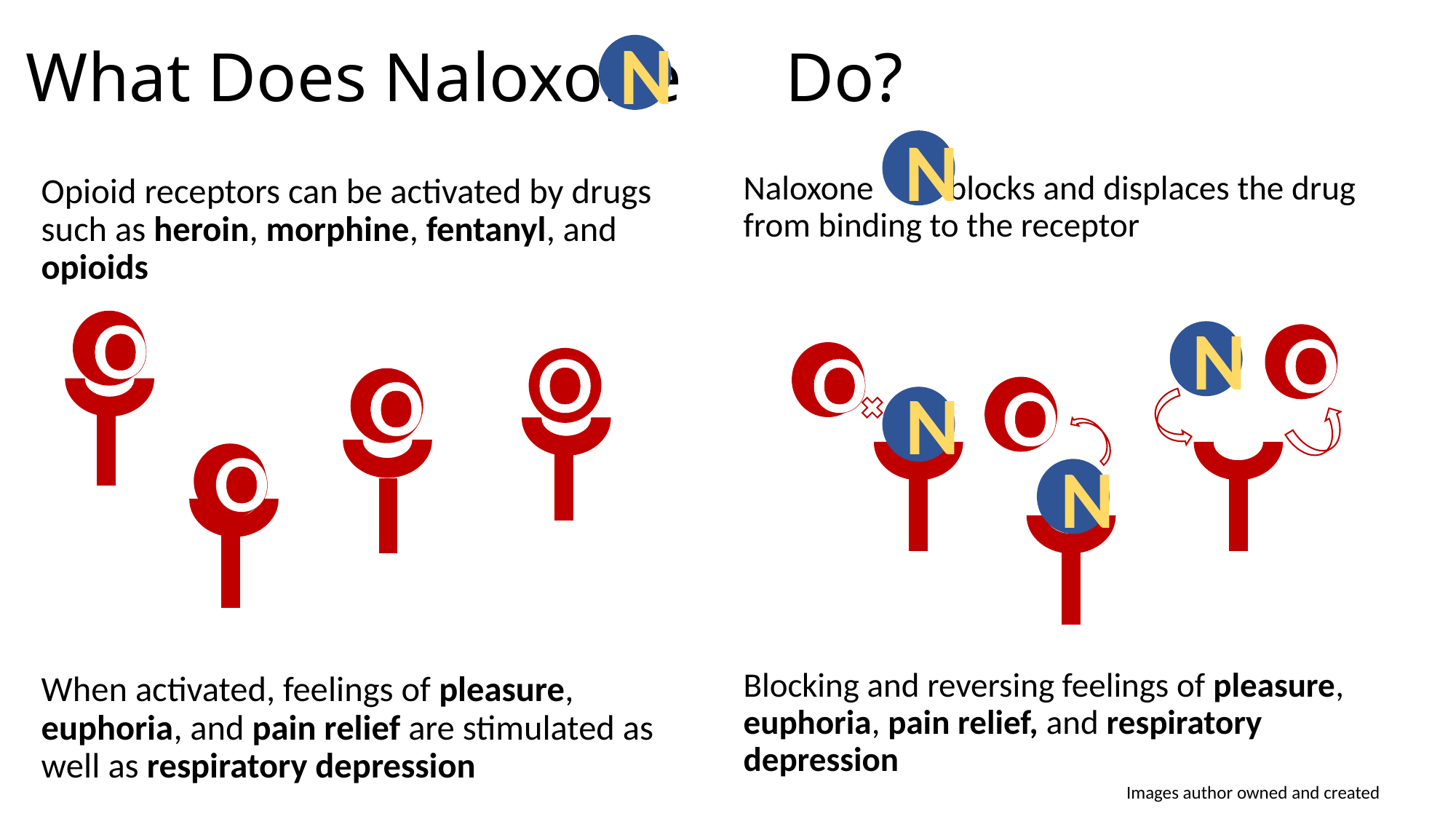

# What Does Naloxone Do?
N
N
Naloxone 	blocks and displaces the drug from binding to the receptor
Blocking and reversing feelings of pleasure, euphoria, pain relief, and respiratory depression
Opioid receptors can be activated by drugs such as heroin, morphine, fentanyl, and opioids
When activated, feelings of pleasure, euphoria, and pain relief are stimulated as well as respiratory depression
O
N
O
O
O
O
O
N
O
N
Images author owned and created

## Slide 3
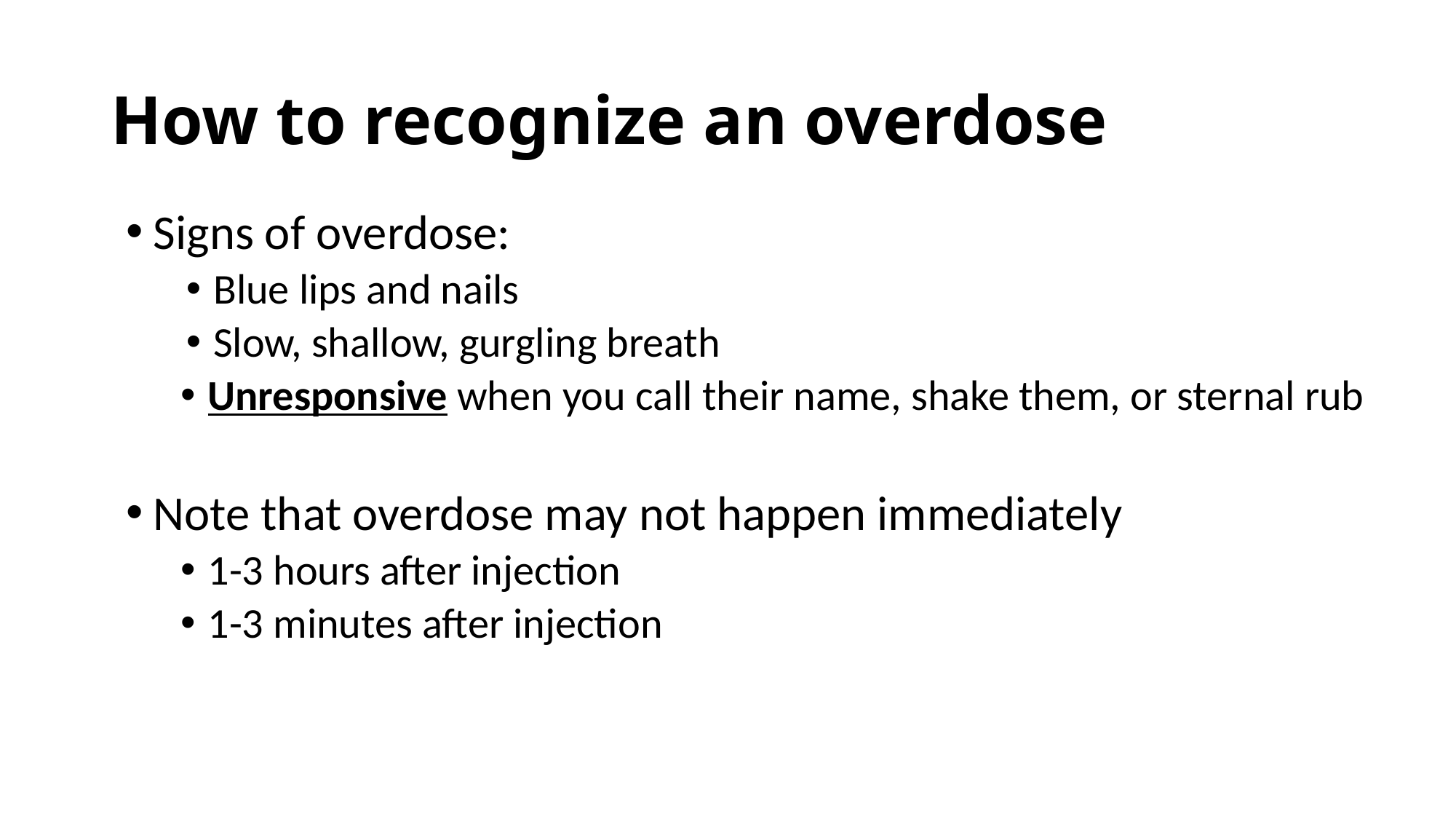

# How to recognize an overdose
Signs of overdose:
Blue lips and nails
Slow, shallow, gurgling breath
Unresponsive when you call their name, shake them, or sternal rub
Note that overdose may not happen immediately
1-3 hours after injection
1-3 minutes after injection

## Slide 4
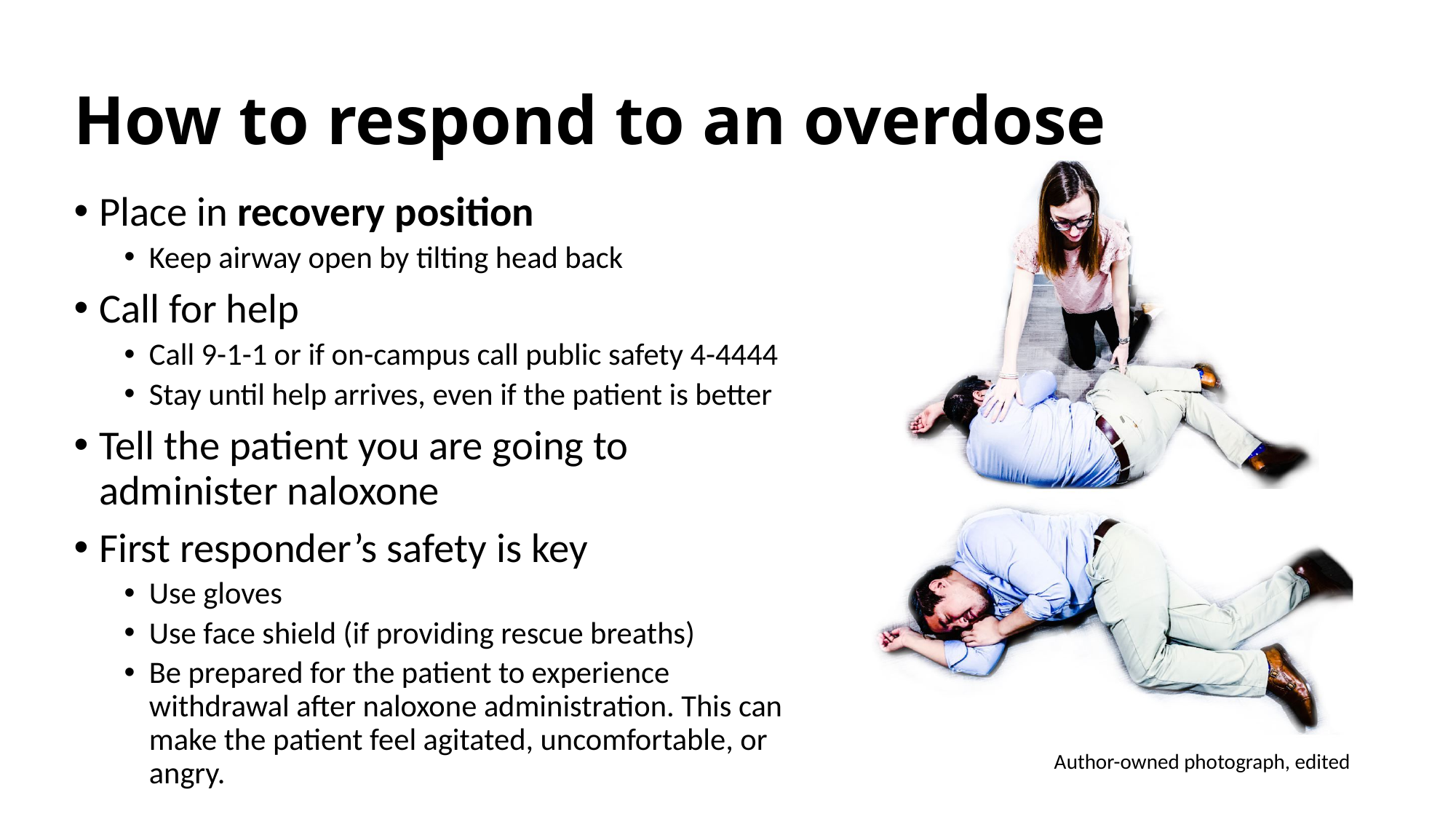

# How to respond to an overdose
Place in recovery position
Keep airway open by tilting head back
Call for help
Call 9-1-1 or if on-campus call public safety 4-4444
Stay until help arrives, even if the patient is better
Tell the patient you are going to administer naloxone
First responder’s safety is key
Use gloves
Use face shield (if providing rescue breaths)
Be prepared for the patient to experience withdrawal after naloxone administration. This can make the patient feel agitated, uncomfortable, or angry.
Author-owned photograph, edited

## Slide 5
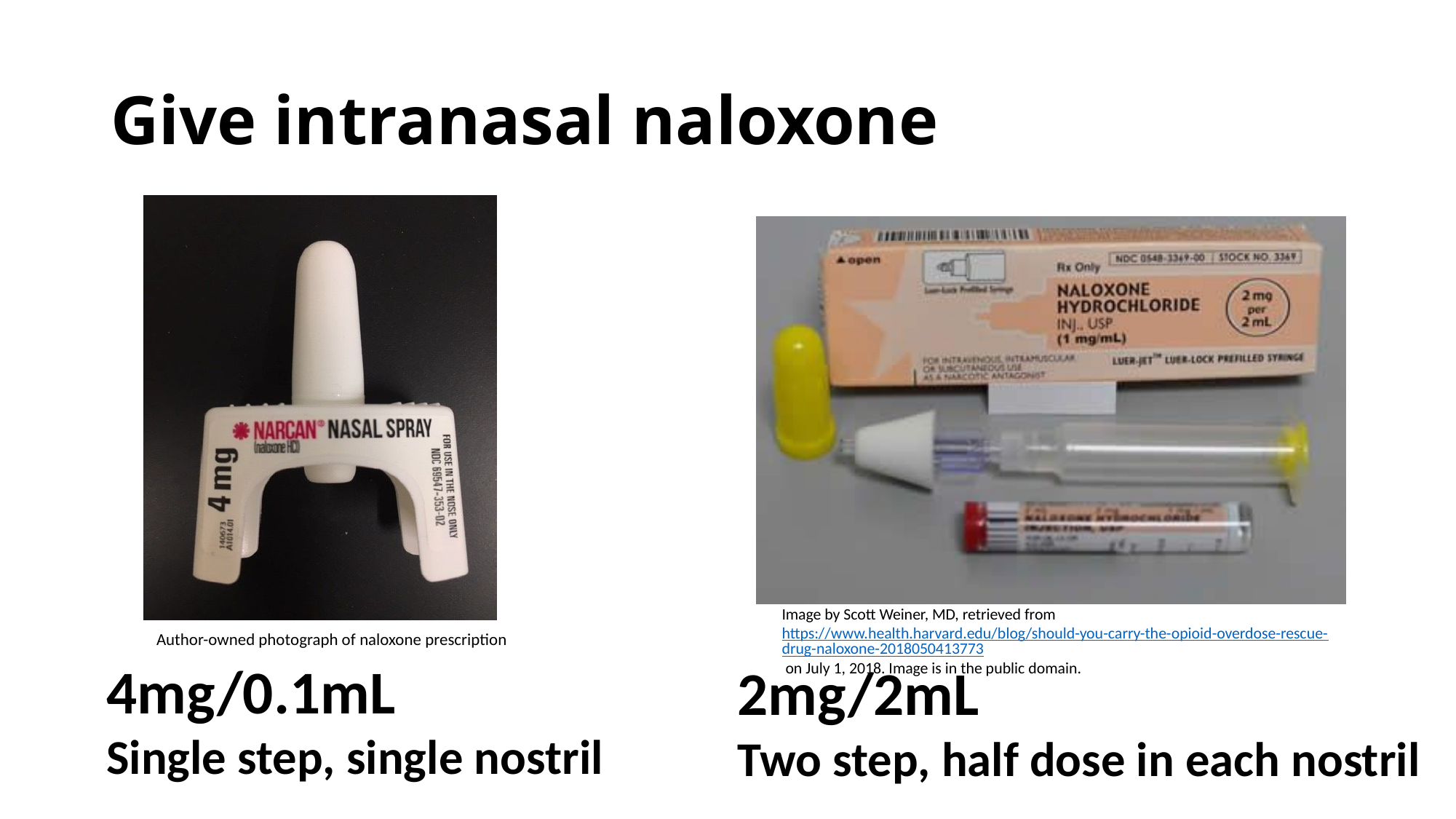

# Give intranasal naloxone
Image by Scott Weiner, MD, retrieved from https://www.health.harvard.edu/blog/should-you-carry-the-opioid-overdose-rescue-drug-naloxone-2018050413773 on July 1, 2018. Image is in the public domain.
Author-owned photograph of naloxone prescription
4mg/0.1mL
Single step, single nostril
2mg/2mL
Two step, half dose in each nostril

## Slide 6
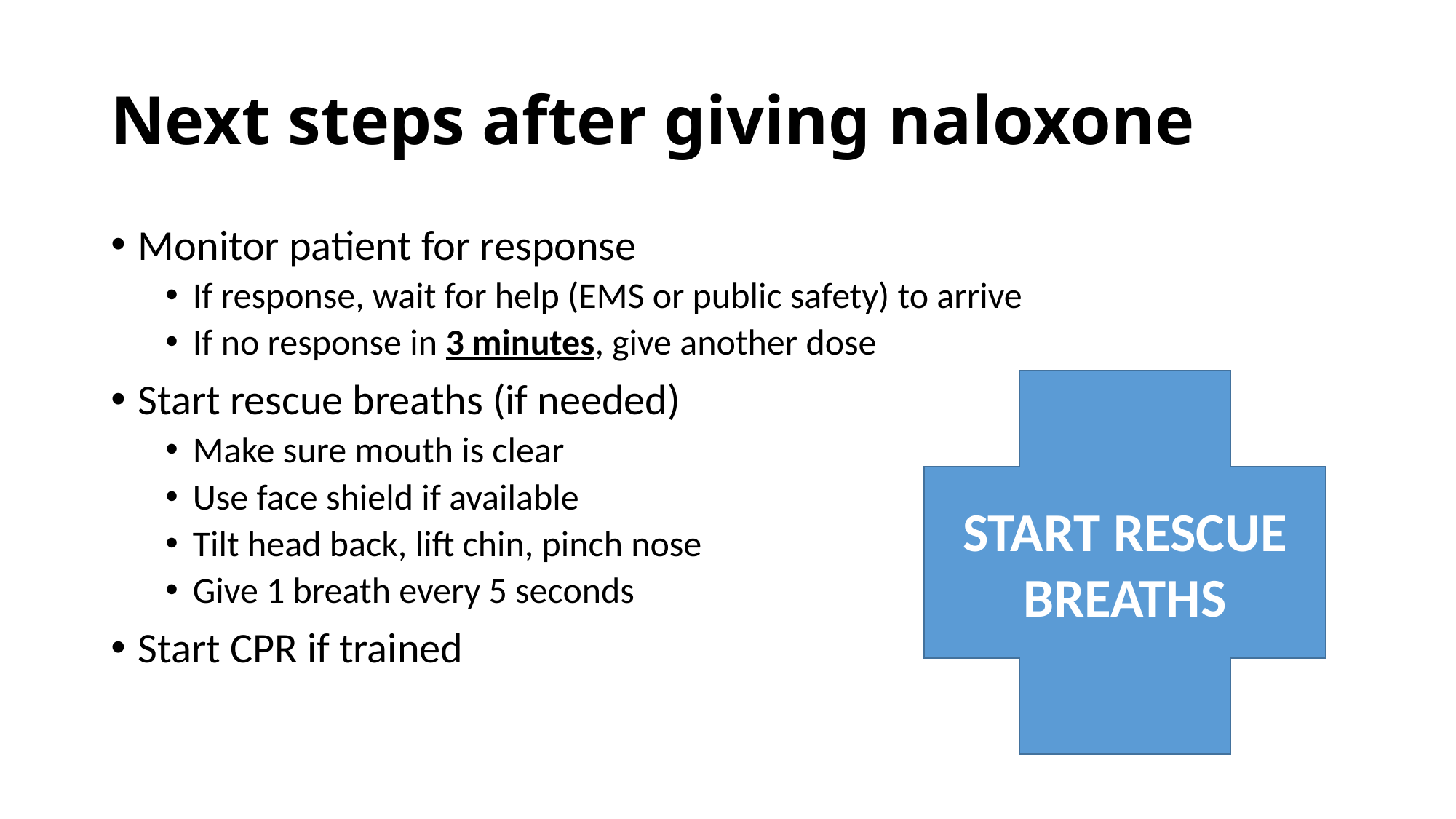

# Next steps after giving naloxone
Monitor patient for response
If response, wait for help (EMS or public safety) to arrive
If no response in 3 minutes, give another dose
Start rescue breaths (if needed)
Make sure mouth is clear
Use face shield if available
Tilt head back, lift chin, pinch nose
Give 1 breath every 5 seconds
Start CPR if trained
START RESCUE BREATHS

## Slide 7
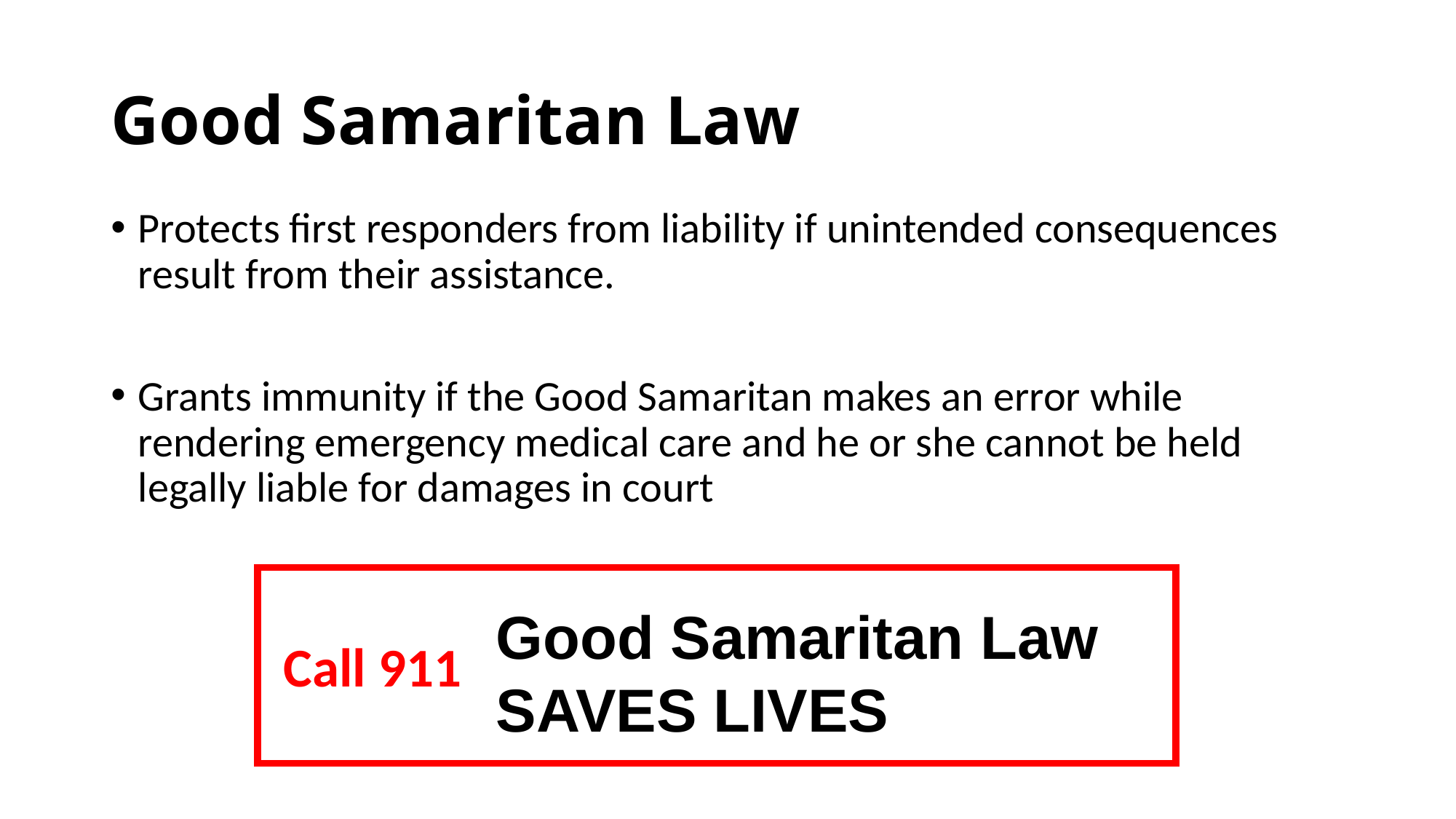

# Good Samaritan Law
Protects first responders from liability if unintended consequences result from their assistance.
Grants immunity if the Good Samaritan makes an error while rendering emergency medical care and he or she cannot be held legally liable for damages in court
Good Samaritan Law
SAVES LIVES
Call 911
