# Supplementary material for: Rapid Naloxone Administration Workshop for Health Care Providers at an Academic Medical Center
Source: MedEdPORTAL. 2020 Feb 14;16:10892. doi: 10.15766/mep_2374-8265.10892 (PMC7062540; doi:10.15766/mep_2374-8265.10892)
Supplement: Supplementary file 1 — A. Naloxone Training Workshop PowerPoint.pptx B. Naloxone Trainer's Guide.docx C. Naloxone Training Video.mp4 D. Training Kit.docx E. Pre- and Postintervention Survey.docx [file mep-16-10892-s001.zip › B. Naloxone Trainer's Guide.docx]

**APPENDIX B: Naloxone Trainer’s Guide**

**Naloxone Training Session:**

**Personnel and materials needed:** Trainer, naloxone training workshop PowerPoint slides (Appendix A), demo single-step and/or two-step naloxone kit

**Objectives**

1. Identify and describe the risk factors for opioid overdose.

2. Describe the clinical presentation of opioid overdose.

3. Demonstrate knowledge of overdose response and correct use of naloxone.

1. **Session Introduction** **(2 minutes)**
   1. Introduce the trainers, including name, title, and department.
   2. Discuss the local opioid epidemic, including local or nationwide statistics of opioid overdose.
   3. Ask participants how naloxone works, and review basic mechanism of action (Slide 2).
2. **How to recognize and respond to an opioid overdose (2 minutes)**
   1. Describe to the participants what an opioid overdose may present as (Slide 3).
      1. Signs of an overdose include blue lips and nails, slow gurgling breath, or unresponsive to sternal rub.
   2. Discuss the steps to complete once an opioid overdose is suspected (Slide 4).
      1. Place the person in the recovery position.
      2. Call for help (911).
      3. Ensure first-responder safety by wearing gloves or using a face shield.
      4. Verbalize to the patient that will be administering naloxone.
3. **Naloxone counseling, including demonstration device (5 minutes)**
   1. Distribute demonstration devices to all participants. Have the participants follow along with the following steps (Slides 5, 6):
      1. Remove naloxone from the foil package. Place the device into one nostril. Press the device plunger.
      2. If needed, start rescue breaths until emergency medical services arrive.
      3. Wait 3 minutes prior to giving another dose of naloxone.
   2. Explain the Good Samaritan Law to ensure that participants are aware of their legal duties and rights (Slide 7).
      1. The Good Samaritan Law grants immunity if the Good Samaritan makes an error when rendering emergency medical care. He/she cannot be held legally liable for damages in court.
4. **Question/answer session (1 minute)**
   1. Ask the participants if they have any questions regarding how to identify an opioid overdose, how to respond to an opioid overdose, and how to administer naloxone.
   2. Encourage participants to fill naloxone prescription at the local pharmacy, to ensure all first responders are equipped with the medication.
