# Supplementary material for: Rapid Naloxone Administration Workshop for Health Care Providers at an Academic Medical Center
Source: MedEdPORTAL. 2020 Feb 14;16:10892. doi: 10.15766/mep_2374-8265.10892 (PMC7062540; doi:10.15766/mep_2374-8265.10892)
Supplement: Supplementary file 1 — A. Naloxone Training Workshop PowerPoint.pptx B. Naloxone Trainer's Guide.docx C. Naloxone Training Video.mp4 D. Training Kit.docx E. Pre- and Postintervention Survey.docx [file mep-16-10892-s001.zip › E. Pre- and Postintervention Survey.docx]

**Appendix E: Pre and Post Intervention Survey**

Please answer the following survey questions below:

**Q1 How comfortable are you administering Naloxone as a first responder?**

[ ] Extremely uncomfortable

[ ] Moderately uncomfortable

[ ] Neither comfortable nor uncomfortable

[ ] Moderately comfortable

[ ] Extremely comfortable

**Q2 How comfortable are you teaching your patients how to administer Naloxone?**

[ ] Extremely uncomfortable

[ ] Moderately uncomfortable

[ ] Neither comfortable nor uncomfortable

[ ] Moderately comfortable

[ ] Extremely comfortable

**Q3 What percent of the time do you currently prescribe Naloxone for your patients with opiate addictions or high risk IV drug use behaviors?**

[ ] 0-25%

[ ] 26-50%

[ ] 51-75%

[ ] 76-100%

**Q4 Are you interested in getting a Naloxone prescription after completion of this training?**

[ ] Yes

[ ] No
