# Supplementary figures and images for: Rapid Naloxone Administration Workshop for Health Care Providers at an Academic Medical Center
Source: MedEdPORTAL. 2020 Feb 14;16:10892. doi: 10.15766/mep_2374-8265.10892 (PMC7062540; doi:10.15766/mep_2374-8265.10892)

**Appendix D: Training Kit**


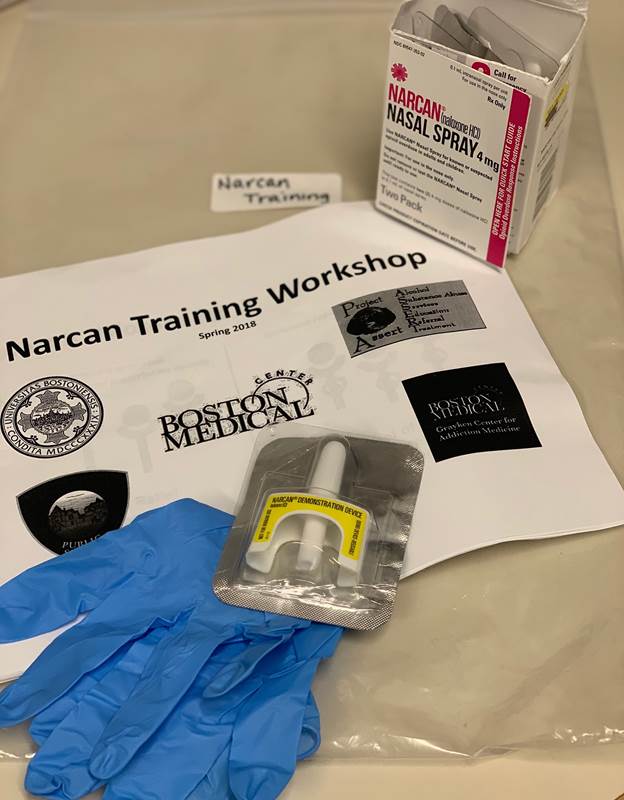

Supplement: Supplementary file 1 — A. Naloxone Training Workshop PowerPoint.pptx B. Naloxone Trainer's Guide.docx C. Naloxone Training Video.mp4 D. Training Kit.docx E. Pre- and Postintervention Survey.docx [file mep-16-10892-s001.zip › D. Training Kit.docx]
